# Listening in the bog: I. Acoustic interactions and spacing between males of *Sphagniana sphagnorum*

**DOI:** 10.1007/s00359-018-1250-8

**Published:** 2018-02-13

**Authors:** Glenn K. Morris, Aaron M. Hall, Heiner Römer

**Affiliations:** 1 Department of Biology, University of Toronto at Mississauga, Mississauga, Canada; 2 Denver, USA; 3 Zoology, University of Graz, 8010 Graz, Austria

**Keywords:** Katydid, Acoustic spacing, Sound transmission, Distance estimation, Aggression

## Abstract

Males of the katydid *Sphagniana sphagnorum* form calling aggregations in boreal sphagnum bogs to attract mates. They broadcast frequency-modulated (FM) songs in steady series, each song comprised of two wing-stroking modes that alternate audio and ultrasonic spectra. NN analysis of three populations found mean distances between 5.1 and 8.4 m, but failed to find spacing regularity: in one males spaced randomly, in another they were clumped, but within the clumps spaced at random. We tested a mechanism for maintaining inter-male distances by playback of conspecific song to resident males and analysing song interactions between neighbouring males in the field. The results indicate that the song rate is an important cue for males. Information coded in song rates is confounded by variation in bog temperatures and by the linear correlation of song rates with temperature. The ultrasonic and audio spectral modes suffer different excess attenuation: the ultrasonic mode is favoured at shorter distances (< 6 m), the audio mode at longer distances (> 6 m), supporting a hypothesized function in distance estimation. Another katydid, *Conocephalus fasciatus*, shares habitat with *S. sphagnorum* and could mask its ultrasonic mode; however, mapping of both species indicate the spacing of *S. sphagnorum* is unaffected by the sympatric species.

**Electronic supplementary material:**

The online version of this article (10.1007/s00359-018-1250-8) contains supplementary material, which is available to authorized users.

## Introduction

Males of most crickets and katydids (Ensifera, Orthoptera) broadcast songs. Diverse aspects of this singing behaviour are much studied: the function and evolution of their hearing organs, the effects of acoustic signal differentiation on speciation, mate choice, aggregation, chorusing, etc. (Bradbury and Vehrencamp 2011; Gerhardt and Huber 2002; Greenfield 2002; Gwynne 2001; von Helversen and von Helversen 1994; Pollack et al. 2016). Generally, when females of these acoustically communicating insects are tested for phonotaxis in both lab and field, they readily approach the male broadcasting the song. This reliable behavioural response has allowed researchers to perform numerous studies on sound localization and pattern recognition (Gerhardt and Huber 2002; Greenfield 2002; Römer 2015; Schöneich et al. 2015).

However, this broadcast signal can also elicit either positive or negative phonotaxis, or aggression in conspecific males (Doolan 1981; Mason 1996). For example, males of the meadow katydid *Orchelimum gladiator* orient to the songs of neighbouring male conspecifics and engage them in fights (Morris 1971, 1972). Males display the same aggressive approach to a speaker broadcasting conspecific song. Aggregations of singing males, within which individuals position themselves at regular inter-male distances, can result from acoustic interactions and corresponding movement toward and away from rivals. Confirming the repulsive role of song, deafened males no longer display acoustic-mediated spacing (Thiele and Bailey 1980; Chamorro-Rengifo et al. 2007). The collective outcome of attraction and repulsion in response to conspecific song can often be regular spacing (Morris 1967; Thiele and Bailey 1980; Meixner and Shaw 1986; Schatral et al. 1984; Chamorro-Rengifo et al. 2007; but see Dadour 1990, and his re-analysis of Schatral et al.’s data.). The results of a multitude of other playback experiments have shown that male song may have functions other than mate attraction, such as functions related to chorusing, satellite behaviour, aggregation, territory, and spacing (Morris 1971, 1972; Thiele and Bailey 1980; Hissmann 1991; Greenfield and Minkley 1993; Bailey and Field 2000; Greenfield 2002; Chamorro-Rengifo et al. 2007).

The sensory cues (signals) that enable individuals to maintain a given distance from neighbouring males in insects, however, are unclear. The ranging hypothesis that was originally developed for distance estimation in birds states that the signal degradation that occurs during transmission, due to factors such as frequency-specific excess attenuation or degradation in the time domain, may represent an effective sensory cue for receivers (Morton 1986; Naguib and Wiley 2001; Morris et al. 2016). Even frogs appear to rely mainly on more complex acoustic cues rather than amplitude alone when assessing the distance to sound sources, such as spectral degradation or reverberation (Ringler et al. 2017). Maximum hearing distances of about 50 m have been reported for katydids, and these are distances over which acoustic signals will have degraded to varying degrees, depending on the physical properties of the transmission channel (Michelsen and Larsen 1983; Römer and Lewald 1992; Römer 1998). Since katydids often use broadband signals that include high sonic and ultrasonic frequencies that suffer from strong, frequency-dependent, excess attenuation, the spectral changes associated with distance could be cues used by males for spacing (Morris et al. 2016).

**Fig. 1:**
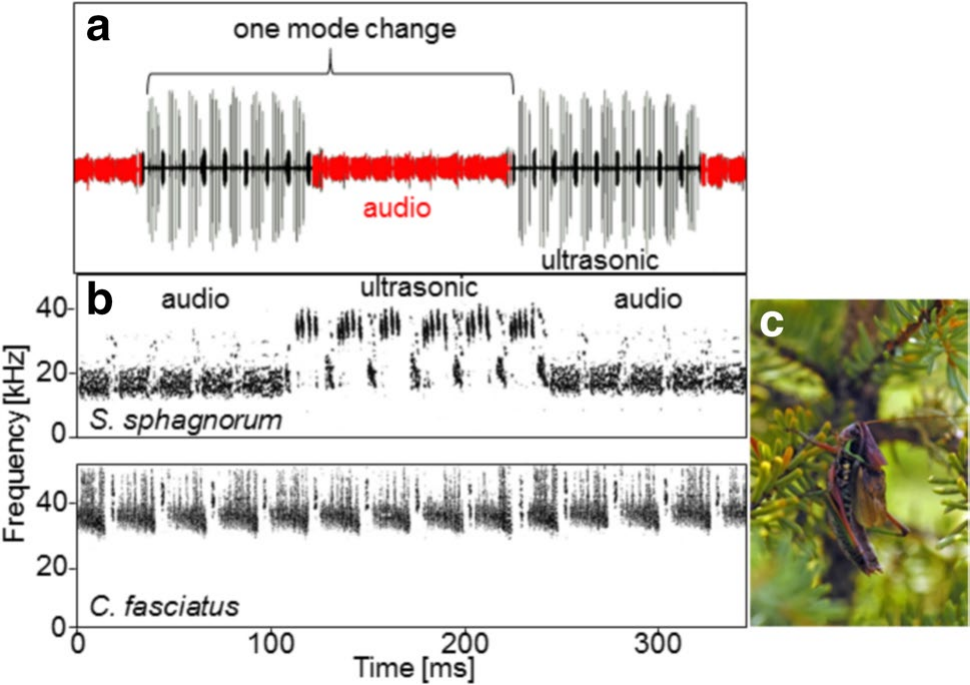
**a** Oscillogram of a section of *S. sphagnorum* song, with the audio- (red) and ultrasonic (black) part as one mode change used to calculate the song rate. **b** Sonogram of *S. sphagnorum* and *C. fasciatus* song (upper and lower panel, respectively). Note that the frequencies of the sympatric species overlap with the frequencies of the ultrasonic, but not the audio mode of *S. sphagnorum*. **c** Photograph of a *S. sphagnorum* male in a typical singing position on a branch of a small spruce tree

Males of *Sphagniana* (formerly *Metrioptera*) *sphagnorum* sing each summer in the boreal forests and bog wetlands of Canada. The species occurs from the Hudson Bay Lowlands northwest to the Yukon (Vickery and Kevan 1985). Neither sex flies and the micropterous females are silent. The brachypterous males perch on ground vegetation, but more often in spruce trees and stridulate singing both day and night. The song of this species consists of two parts that differ in their pulse structure (time domain) and carrier (frequency domain) (Fig. 1a; see also supplementary video S1, and Morris 1972, 2008). Whereas, one part is dominated by high-audio frequencies with maxima close to 17 kHz, the other part has its maximum energy in the ultrasonic range near 35 kHz (Fig. 1b). The ultrasonic spectrum arises from short, sinusoidal, high-Q pulses produced by a stick–slip mechanism (Patek et al. 2011) that involves distortion-based elastic energy (Montealegre et al. 2006). Uninterrupted series of changes between these two song modes are produced by males for several hours. Although uncommon, such frequency modulation has been reported for another katydid (*Xiphidium amplipennis*; Morris et al. 2016) and for a group of crickets belonging to the genus *Eneoptera* (Eneoperinae), which switch carrier frequencies from about 4–15 kHz (Robillard and Desutter-Grandcolas 2011).

Another katydid, *Conocephalus fasciatus* (the slender meadow katydid), often shares its habitat and communicates simultaneously with *S. sphagnorum. C. fasciatus* individuals occur at a much higher density. They alternate a sustained buzz with a long series of ticks and emit songs with a spectrum that includes an intense ultrasonic band of 28–80 kHz (Fig. 1b; Morris 1999). Calling from their positions on grasses and low ericaceous shrubs just above the sphagnum moss surface, members of this species appear to present a masking problem for individuals of both sexes of *S. sphagnorum* as the latter attempt to listen and localize conspecific calls. However, given the ultrasonic nature of the song, we hypothesized that the song of *C. fasciatus* could effectively mask only the ultrasonic, but not the audio, mode of *S. sphagnorum* song.

In this first of a series of two papers, we describe the spacing behaviour and acoustic interactions observed among males in the field as well as how resident males responded to playbacks that simulated the songs of rival males. In most other katydid species signal attenuation affects all song components equally, but we predicted that the two alternating, high-audio and ultrasonic modes in the song of *S. sphagnorum* would be differently affected by frequency-dependent excess attenuation. Thus, we quantified the degree of frequency-dependent attenuation for the two song modes, which could be used by receivers as a cue for ranging. In the second paper, the representation of *S. sphagnorum* song in the afferent auditory pathway is described, and how this insect might distinguish closer from more distant singing males.

## Methods

### Study sites

Field sites were located 150 km northwest of Lake Superior near the watershed divide of the Arctic and Atlantic Oceans. Here the insects form populations in peat landforms classified as ‘flat-bog wetlands’ (Warner and Rubec 1997). In contrast to the surrounding dense, mature, spruce forest, the flat-bog habitat is meadow-like and acoustically open. Between scattered clumps of stunted (< 4 m high) spruce are spaces carpeted with a thick, hummocky layer of highly sound-absorbent vegetation: sphagnum moss, grasses, sedges, and low-growing, finely branched, woody, ericaceous shrubs (see supplementary photograph S2). A *S. sphagnorum* male is often seen singing from terminal spruce shoots, though they occasionally sing from the sphagnum carpet. *C. fasciatus* never perch on the trees, but form dense demes that do not exactly coincide with those of *S. sphagnorum*.

Measures of horizontal nearest neighbour distances (NND) between singing males were obtained at two sites, designated Niblock (49°5.4′N and 90°41.55′W) and Trewartha (49°8.035′N and 90°46.213′W). At the Niblock site, populations of both species that occupied an embayment off a much larger bog were mapped. At the Trewartha site, although both species were present, we obtained NND measures only for *S. sphagnorum* males.

### Perch heights and spacing

#### Niblock population

**Fig. 2:**
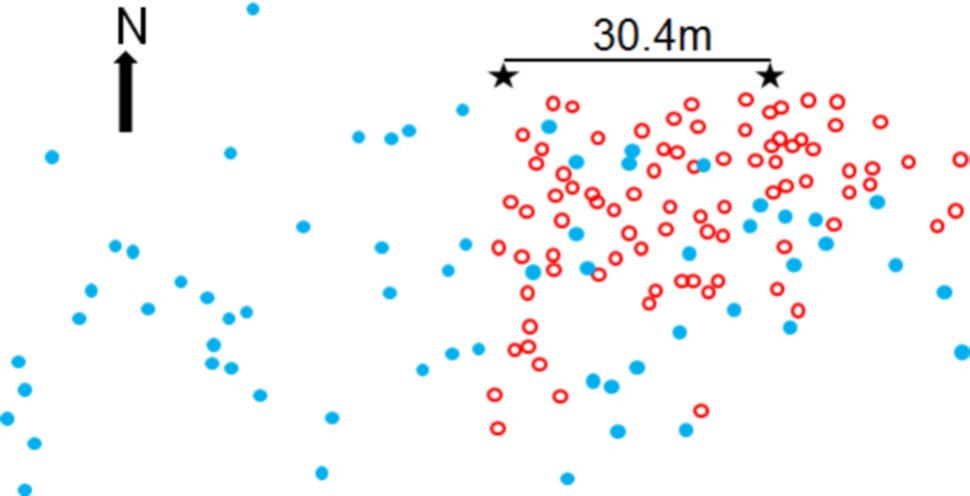
Map of the spatial distribution of singing sites in the Niblock population for *S. sphagnorum* (blue) and *C. fasciatus* (red) males. The asterisks mark the 30.4-m baseline used for theodolite bearings. Mean NND of 2.5 m for *C. fasciatus* contrasts with 5.1 m measured for *S. sphagnorum*

Singing males were localized using a heterodyne ‘bat detector’ (Ultrasound Advice U30) taped to the handle of a fishing rod with the microphone located at the rod tip. Each singer was localized by advancing into the population area and moving the fishing rod along incremental parallel transects, using the high directionality of the device and the strong sound gradients. The heterodyne converts insect ultrasound into an audio signal that is audible via earphones; the rod-mounted microphone enabled rapid tracking of the singers. This scanning census was first conducted with *S. sphagnorum*, and on the subsequent day with *C. fasciatus*. Although the census interval of one day did not allow a more detailed analysis of the association between individuals of *S. sphagnorum* and *C. fasciatus*, the different distribution of *C. fasciatus* in the eastern and western part of the *S. sphagnorum* population (Fig. 2) did not change when we mapped *C. fasciatus*. The scanning process occurred without disturbing the singing of males greatly; they continued singing or shortly after the scanner had passed by. Although not required for the mapping, *S. sphagnorum* males were frequently seen, while *C. fasciatus* males were seen less frequently. Localization using the sound gradient was accurate within a range of about 20 cm. Populations of the two species were flagged with different colours.

After mapping all males, we used an optical theodolite (Micrometer T1 Wild) to obtain a pair of angles for each marked singing perch. The theodolite was supported on a tripod that had been placed on a plywood platform that was built in the bog atop tree stumps. From first one end and then the other of a 30.4-m baseline, sightings were made to a standard that was held successively at each flagged singing location. Using the baseline as an axis, we obtained a pair of angles for each male, the degrees of which we later converted to radians. The baseline and angles were the basis of the constructed map (Fig. 2).

For the mapped Niblock populations, we performed a nearest neighbour analysis in Esri ArcView 10.1 (esri.com). This analysis calculates the distance between each individual and its nearest neighbour, then compares these distances against the expectation that the individuals are randomly distributed. If a significant negative Z index is obtained, this indicates that individuals are clumped more closely together than would be expected in a randomly distributed population, while obtaining a significant positive value indicates that the individuals are more regularly spaced.

#### Trewartha population

At the Trewartha site, two populations of *S. sphagnorum* were studied in two subsequent seasons; no attempt was made to establish a map of males. In one season we marked a 75-m baseline along a north-running access road. From each end of this baseline, a row of stakes was driven every 10 m while advanced eastward into the bog to delimit a large, rectangular area that encompassed all the *S. sphagnorum* population therein. Heterodyne scanning (as above) was conducted in the mid-afternoon and took about 3 h to complete. Each singing male was located and flagged. On the next day, the nearest neighbour distances were obtained using direct tape measurement. We performed the Chi-square test (Campbell and Clark 1971) on these NND data to examine the goodness of fit. This test is used to assess the distribution of nearest neighbour distances as compared to an expected random distribution. We also performed the Pielou’s analysis (Pielou 1962) on a truncated set of NND data, which allowed us to test the spatial distribution within clumps if these were found to be present in the Campbell and Clark test.

### Field measures: song rates, perceived intensities, attenuation, and temperature effects

In the following season, we examined song interactions between two neighbouring males in the field by measuring the rate of the mode changes in the songs. From a distance that would not disturb the insects during singing, we recorded a time sample (~ 1 min) of each male’s calling song using a microphone (Sony ECM909) connected to a Walkman (Sony) tape recorder. This audio-limited equipment was sufficient to record the frequencies in the audio mode of the song. From these recordings, we later determined the rate of mode changes in the songs of each male and the differences in these rates between the two males as a function of their distance from one another. From these data we also calculated the mean nearest neighbour distances for this population.

We also obtained a measure of the perceived intensity of the song of the nearest neighbour (dB SPL—RMS Fast reading) at a focal male´s position. The perceived intensity of one neighbour was measured from the perch of the other. A microphone (1/2″, Type 4133, Bruel and Kjaer) connected to a precision sound level meter (B&K Type 2209) was held at the position of the focal male (which silenced his singing) and pointed in the direction of his neighbour, who continued to sing. These values provided realistic measures of the sound intensities at which each singer would perceive his neighbours in the field. Subsequently, the distance between the pair of males, their perch heights, and the heights of the trees were recorded.

To determine the amount of attenuation in the audio and ultrasonic modes within the song of *S. sphagnorum*, we broadcast a pre-recorded song of a single male in the Trewartha bog along a transect of 20 m, using a Racal 4D instrumentation tape recorder (speed at 38.1 cm/s) and a speaker (Technics EAS-10 TH400A) mounted at a height of 1 m. The broadcast song was recorded at various distances using a Bruel and Kjaer microphone (4133; height 1 m) and pre-amplified with a precision sound level meter (2209) that was equipped with a custom-built, high-pass filter set at 1 kHz. We noted the reading on the sound level meter at several distances for the audio and ultrasonic modes. The broadcast song was also recorded using a heterodyne ‘bat detector’ (Ultrasound Advice U30) connected to headphones, so that a given SPL reading obtained with the sound level meter could be unambiguously assigned to either of the two modes. To obtain a higher signal-to-noise ratio for the recordings at greater distances, the broadcast SPL was set at 102 dB SPL for the audio mode, which is 10 dB higher than the average SPL of a singing male. The broadcast *S. sphagnorum* song was occasionally masked by singers of the sympatric species *C. fasciatus*, and these had to be silenced. Since the calling activity of *S. sphagnorum* males could also interfere with the recording of the pre-recorded song, we established the transect along the borders of the *S. sphagnorum* population, rather than directly within the population, at least 20 m away from all calling males. A total of four transects were examined in this way.

Since changes in ambient temperature affect the gross- and fine-scale temporal properties of the insects’ acoustic signals, we measured the air temperatures on a fine sunny day within the bog habitat. Five thermocouples (Type T) were placed, one at the bog surface and others at various heights on a 2-m high spruce. We made readings over a ~ 25-min period by switching between the channels of a microprocessor-controlled thermometer (Omega Engineering, Model HH23) with the attached multi-probe switchbox (HH-20-SW). This process was repeated with five different trees.

We also determined the dependency of the rate of mode changes on temperature by recording songs from males that were isolated from other males by 8 m or more, so that it was highly unlikely that changes in the song rate were the result of acoustic interactions between males. Using an ECM909 microphone connected to a Sony Walkman tape recorder, we recorded the song of a male for 1 min and subsequently measured the temperature at the singing site of this male using a thermocouple device (TE 1100, OK Electronic). The ambient temperature ranged between 18 and 33 °C during these measurements, due to different climatic conditions, times of the day, sunny or shady conditions, or the height of the males above the bog surface (*N* = 30 males).

### Playback

The song of an isolated, caged *S. sphagnorum* singer was recorded at a distance of 15 cm, using a sound level meter Bruel and Kjaer 2204 fitted with a ¼″ B&K microphone (4135), and processed with a computer-mounted digitizing board (Keithley Instruments DAS 50 PC, 12 bit) at a rate of 200 kilosamples/s. This recording was used in playback experiments conducted with resident males in the field. The playback signal had a song rate of 92 songs/30 s.

The field broadcast was transmitted using a PC-operated digitizing board (Keithley Instruments PCIP AWFG, 12-bit; sampling rate 100 kHz) via a car radio amplifier (Pioneer) to a Panasonic leaf tweeter (Panasonic Technics EAS-10TH400B). This playback system delivered frequencies with a flat frequency response of up to 50 kHz and, thus, also included most of the energy in the ultrasonic mode. The sound pressure level of the playback, as measured 10 cm from the speaker, was set to 97 dB for the ultrasonic mode and 92 dB for the audio mode [measured with the B&K 2204 sound level meter above-mentioned (1/4″ microphone on Impulse)]. At distances of 1.5–2.0 m from a tested singer, these levels were about 70 and 65 dB, respectively.

The tweeter was clamped to a metal rod and placed 1.5–2.0 m from a singing male with its horn directed at the insect. Each experiment was conducted for a 15-min duration: first, a 5-min sample of undisturbed singing from the male was recorded. Then, without ceasing recording, the song was broadcast from the speaker for 5 min. After the playback had stopped, the focal male’s song was recorded for another 5 min. During the experiment, the test insect’s behaviour was noted by one author while another recorded the insect’s song using a Sony Walkman tape recorder and ECM909 (Sony) microphone fastened to the end of a fishing rod. Taking advantage of the directionality of the microphone, the insect’s call dominated the resulting recording over stimulus broadcast. The songs of thirty-seven males were successfully examined in this way. For each male, we determined the rate of mode changes in the song (per 30 s) for the three 5-min intervals, before, during and after the playback.

We first treated all males as one group and tested for a change in song rate in response to playback, i.e., song rate before playback subtracted from song rate after playback (likewise for ‘during less after’ and ‘before less after’). The null hypothesis states that the singing of a neighbour is ineffective, which would mean that the mean song rates should remain unchanged over time. We conducted three one-sample *t* tests with the collected data of all males in the group, correcting probabilities by following a sequential Holm–Bonferroni procedure to assess whether the mean shifts in the song rate equalled zero under these three conditions.

However, when one group of males increases, the other one decreases its song rate during playback (as was the case), the net result will be no response at all, giving a wrong impression about the effect of playback. Therefore, the cohort of 37 males were separated into two groups based on whether they increased (*n* = 21) or decreased (*n* = 16) their song rate during playback. By ignoring the direction of the rate changes, we analysed changes in the absolute values of rate changes. Each group was analysed separately using a one-way ANOVA (analysis of variance) with repeated measures. To determine the dependent variable song rate, we tested for statistically significant differences between the means of the song rates based on the time factor (before, during, after the playback).

## Results

### Spacing and singing heights

Figure 2 shows a map of the distributions of both male *S. sphagnorum* and *C. fasciatus* individuals in the Niblock bog embayment. It illustrates how individuals of *S. sphagnorum* and the sympatric (and potentially masking) species *C. fasciatus* are distributed. Individuals of *C. fasciatus* clustered in a notable way; all 85 mapped individuals clustered together in the more eastern part of the bog. No male occurred west of the asterisk marking the end of the sightings baseline (Fig. 2). The results of the nearest neighbour distance analysis of the spatial distribution of the 66 singing males of *S. sphagnorum* (using Esri ArcView) (Table 1) indicated that this did not differ from a random distribution. The Z index departed from 0 in a positive direction (regularity) but was not significant (*Z* = 0.761, *p* = 0.447). The same was true for the results of the spatial distribution of *C. fasciatus*. There was a positive but statistically insignificant Z index (*Z* = 0.573, *p* = 0.566). When the data from the two species was combined, no specific spatial patterns in their distributions could be detected (*Z* = −1.095,* p* = 0.274). The mean NND for *S. sphagnorum* in this population was 5.1 m, and that of *C. fasciatus* was 2.6 m. The disparate eastern-western distribution of *C. fasciatus* allowed us to test the null hypothesis of no effect of singing *C. fasciatus* males on the NN distances of *S. sphagnorum*. We found no difference (Wilcoxon rank sum test; *p* = 0.3155), suggesting that there is no substantial masking effect due to singing of *C. fasciatus*.

**Table 1:** Mean NN distances, densities, dispersion indices of *S. sphagnorum* and *C. fasciatus*, compared with other katydids, *O. gladiator, C. nigropleurum*

Species and location	Singers	NN dist. (m) Mean, ± SD	Area (m^2^)	Density (singers/100 m^2^)	Indices *Z, R, χ* ^2^
*S. sphag*. Niblock ON, Canada	66	5.10 ± 0.420	5150	1.282	*Z* = 0.761, *p* = 0.45, not sig.
*C. fasc*. Niblock ON, Canada	85	2.48 ± 0.167	1748	4.863	*Z* = 0.573, *p* = 0.57, not sig.
*O. glad*. NY State USA	117	1.70 ± 0.585	780*	15.0	*R* = 0.318, *p* < 0.01
*C. nigro. NY State, USA*	327	0.90 ± 0.018	780*	41.92	*R* = 1.167, *p* < 0.01
*S. sphag*. Trewartha ON, Canada	101	8.63 ± 0.386	Not applic.	Not applic.	*χ*^2^ = 17.06, *df* = 5, *p* = 0.004
*S. sphag*. Trewartha ON, Canada	57	6.43 ± 0.49	Not applic.	Not applic.	Not applic.

At Niblock *C. fasciatus* overlapped within a more broadly distributed population of *S. sphagnorum*; in NY *C. nigropleurum* and *O. gladiator* both occupied homogenous sedge habitat. For both bog species at Niblock spatial distribution among conspecifics was random; for one population of *S. sphagnorum* at Trewartha there was significant clumping. For both NY species spatial distribution is significantly regular. Niblock density estimates on areas determined by minimum convex hull; NY area* adjusted to include only sedge

Differences in singer densities of the two species were calculated at Niblock. The area occupied by each population was estimated using the minimum convex polygon (MCH) method (smallest polygon incorporating all points). The male density was much higher for *C. fasciatus* with 4.9 males per 100 m^2^ than *S. sphagnorum* with 1.28 males per 100 m^2^.

At the Trewartha site, 101 singing males of *S. sphagnorum* were located, and their positions marked. The mean NND in this population was 8.4 m. Interestingly the only two males found during this census spaced < 1 m from one another were engaged in a (brief) body-contact prolonged audio mode interaction. A Pearson’s Chi-square test was applied to data gathered for this population to examine goodness of fit, and the results showed that the distribution of the individuals deviated significantly from a random distribution (*Χ*^2^ = 17.06, *df* = 5, *p* = 0.004). More individuals than expected were observed spaced at shorter distances from one another, indicating that a clumped distribution existed. Within the clumps identified, running Pielou’s analysis (Pielou 1962) did not allow us to find deviations from random distribution, indicating no additional spatial pattern existed within the larger clumps. In the following season, NNDs of another population of *S. sphagnorum* were analysed at Trewartha. The mean NND in this population was 6.4 m.

**Fig. 3:**
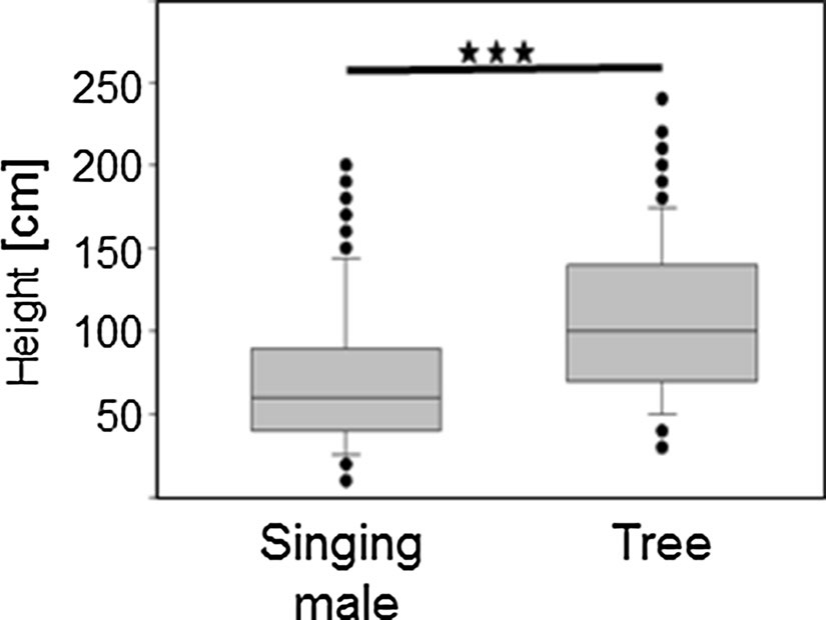
The height of singing males in spruce trees, compared to the absolute height of the occupied trees (*N* = 75)

In the bog substrate used by *S. sphagnorum* the stunted spruce trees stand among an understorey of multiple woody-stemmed shrubs and hummocks of sphagnum (see a typical photograph of the Trewartha site in S1). Males prefer to sing from all of these elevated positions, but mostly from small spruce trees. By contrast, *C. fasciatus* males almost never perch in the trees. The average singing height of *S. sphagnorum* males was 69.8 cm, whereas the average height of trees used by territorial males was 107 cm (Fig. 3), indicating that males did not climb to the maximum available heights to broadcast their songs (Mann–Whitney Rank Sum Test; *N* = 75; *p* < 0.001). The perch heights of individuals of this species were measured over a range from the bog surface (only five males) up to about 2 m. In another survey of *S. sphagnorum* during which only the height of singing males was recorded, the mean perch height of individuals in trees was 53 cm.

### Song interactions and perceived sound pressure levels from neighbours

**Fig. 4:**
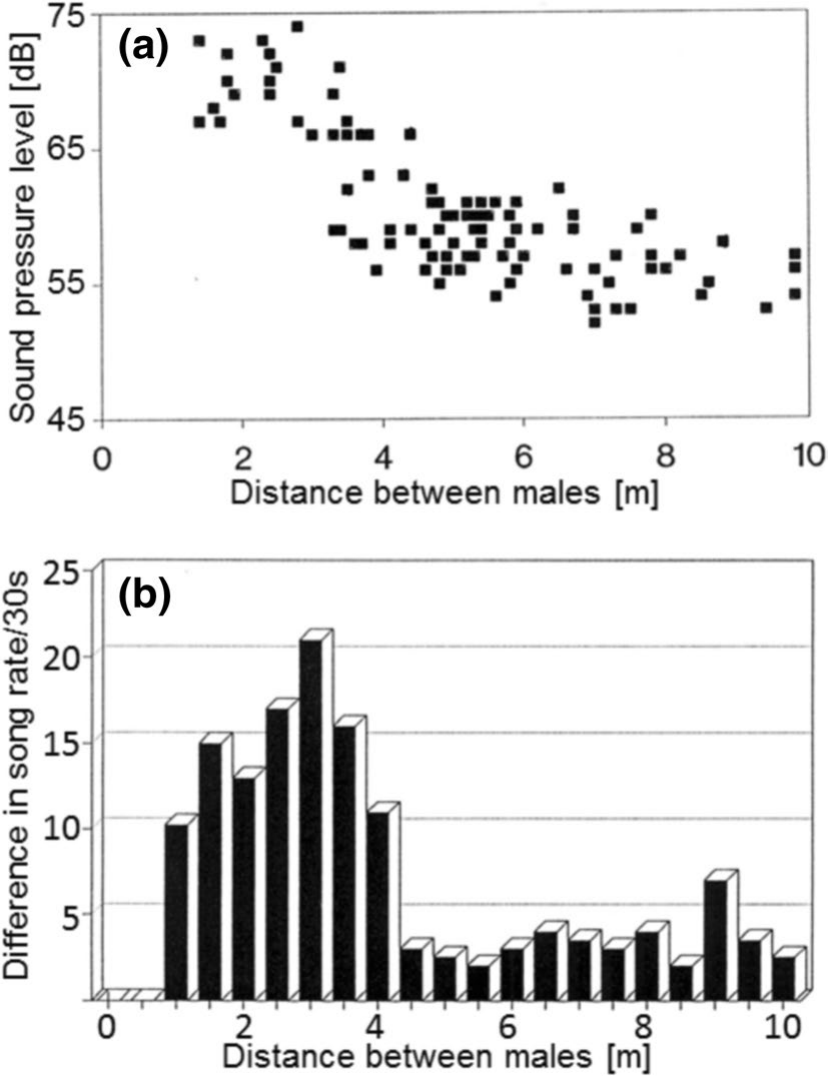
**a** Perceived sound pressure level of the song of the nearest neighbour at the position of a focal male. **b** Analysis of the difference in the rate of song changes between neighbouring males, separated by different distances. Note that significant differences occur when males are < 4 m from one another

The overall SPL of the call of a neighbouring male as perceived by a focal male varies with the inter-male distance (Fig. 4a). (During this measurement, no attempt was made to differentiate between the SPLs of the two song modes.) Approaching the singer there was only a moderate increase in the SPL from 10 to 5 m, but then the SPL increased more steeply to ~ 70 dB at 2 m. The results of an analysis of the song rates of nearest neighbour males indicated that those separated by more than 4 m had similar song rates, which differed on average by only 2–3 song changes/30 s (Fig. 4b). At distances of 4 m and less, however, one male of the pair sang at a much higher rate than the other, resulting in differences of 10–20 song changes/30 s.

**Fig. 5:**
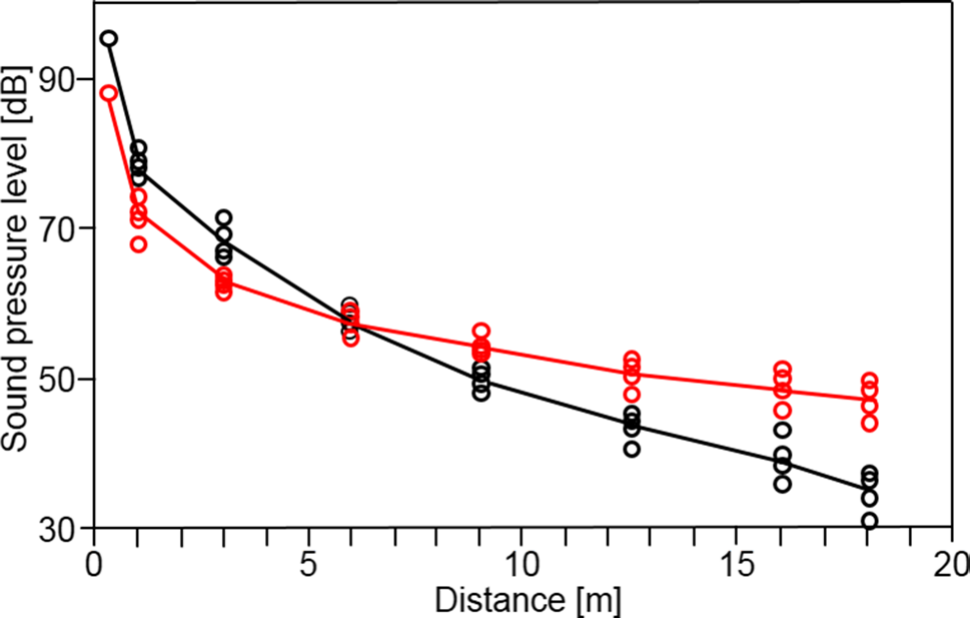
Attenuation of the audio (red) and ultrasonic modes (black) over a transect in the Trewartha bog habitat of *S. sphagnorum*. Note the stronger excessive attenuation of the ultrasonic mode

In the habitat of the Trewartha population, the two song modes show different attenuation over distance (Fig. 5). Whereas, the ultrasonic mode was 7 dB more intense at close range (distance 0.5 m), both modes had equal SPLs at 6 m, and at distances greater than 6 m, the audio mode dominated the signal. At a distance of 15 m, the SPLs of the audio and ultrasonic modes were 48.5 and 39.5 dB, respectively. Compared to geometric spreading, the amount of excess attenuation for the two modes at 15 m was about 10 dB for the audio mode and 26 dB for the ultrasonic mode.

**Fig. 6:**
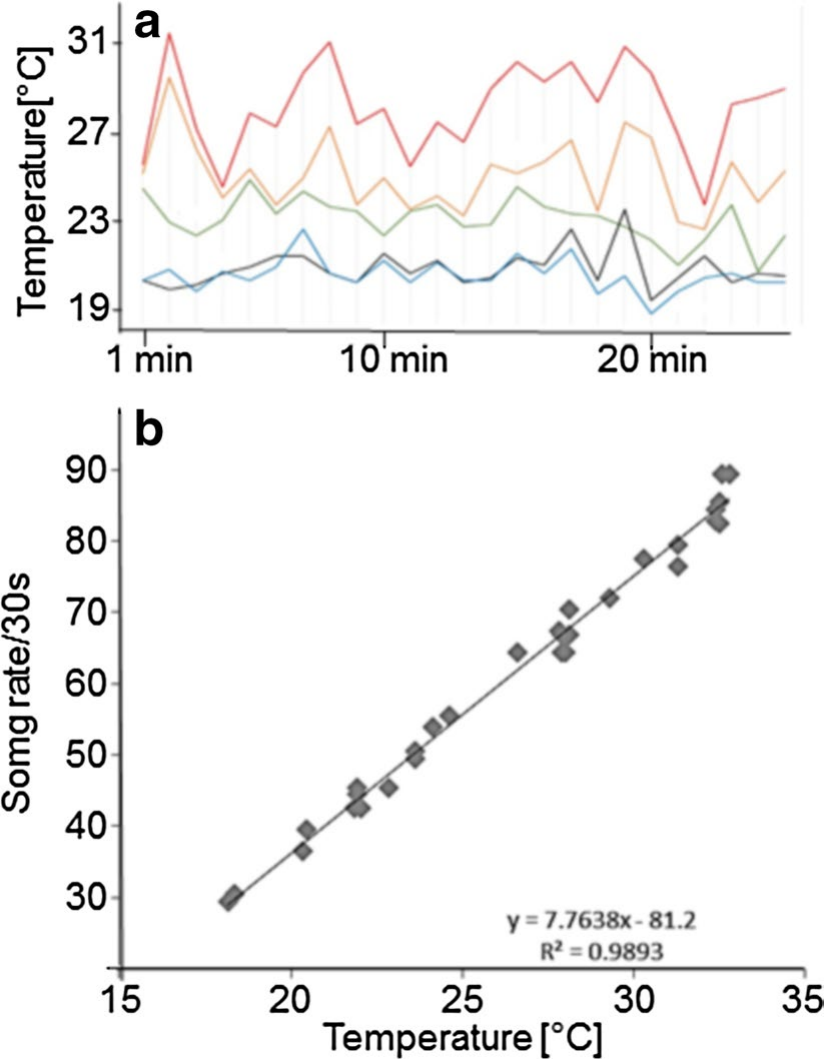
**a** Result of continuous measurement of temperatures over 26 min at different heights in a sphagnum bog under conditions of nearly constant full sunshine. Red: 1 cm above sphagnum surface; orange: spruce branch at height of 26 cm; green: spruce branch at height of 46 cm; black: height of 1.5 m, 26 cm from the trunk; blue: 1.5 m from the trunk. Note that surface temperature consistently exceeded those measured higher up in the tree. **b** Dependency of the rate of mode changes of males on ambient temperatures (*N* = 30)

Temperature has a strong effect on the parameters of the calling songs of ectothermic insects (Walker 1962; Doherty 1985; Gerhardt and Huber 2002; Symes et al. 2017). Our measurements of air temperatures under conditions of steady sunshine at different heights within the bog vegetation revealed a strong warming effect on the sphagnum surface (Fig. 6a). Under conditions of intense solar radiation, the air at the sphagnum moss surface was always substantially warmer than the air in the tree branches at different heights (e.g., at 46 cm or at 1.5 m above the ground surface). On average, the temperature was 7.5 °C higher (range 3.8–10.8) at the surface than at typical perch heights measured in spruce trees.

We conclude that these temperature differences contribute to differences in the song rates displayed by males calling from the bog surface and males calling higher up. We examined variations in the ambient temperature that resulted from the influences of general weather conditions, sunny and shady conditions, or the position of singing males above the bog surface to determine the dependency of mode rates upon temperature (Fig. 6b). The total range of temperatures during these measurements was between 18 and 33 °C, and within this range a strong, linear positive relationship (*R*^2^ = 0.9893) with the mode rate was observed, which increased from about 30 modes/30 s at 18 °C to about 85 modes/30 s at 33 °C.

### Response of males to broadcast song

**Fig. 7:**
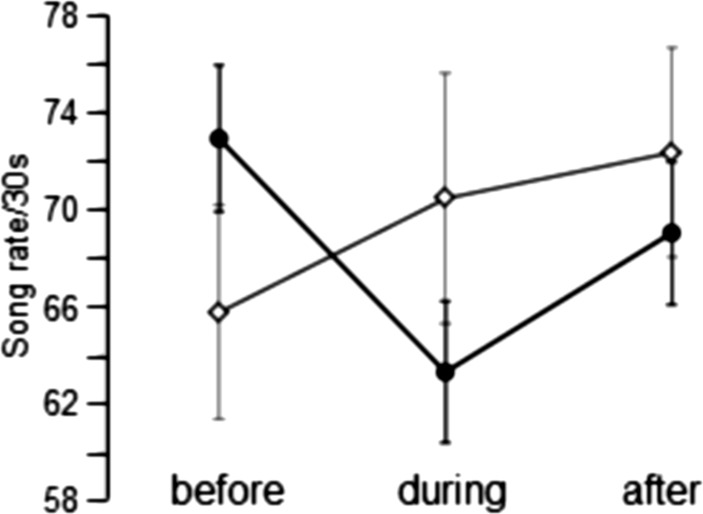
Result of playback experiments in which conspecific songs were broadcast to resident males. Mean song rates before, during, and after the playback for males that either increased (open diamonds) or decreased (filled circles) their song rates. Values are means ± SEM

Broadcast of conspecific calling song in a territorial context can initiate positive phonotaxis in katydids (e.g., Morris 1972). Therefore, we anticipated that *S. sphagnorum* males might approach or withdraw from the speaker as conspecific songs were broadcast (display positive or negative phonotaxis), since such playback may simulate the presence of an intruder into the territory of the resident male. However, we observed no directed movement because of playback; the tested males remained perched in the trees into which they had climbed and continued singing over the course of the 15-min protocol. However, their song rate was affected by these playbacks. About half of the 37 singers (*n* = 18) increased their song rate during playback, and almost as many (*n* = 16) decreased their rate; only 3 did not change their song rate. During the playback, the increases in the song rate ranged from 1 to 19 mode changes/30 s, and the rate decreases ranged from 1 to 27 mode changes/30 s (Fig. 7).

After playback had ended, the song rates of 23 out of 37 males had increased, the rates of 12 had decreased, and the rates of 2 remained unchanged. Rate increases on playback cessation ranged from 1 to 22, with 10 insects increasing their singing by > 5 songs/30 s. Of the eighteen singers who had increased their singing rate during playback, eight increased their rates still higher during the post-playback interval after the stimulus had been removed; nine reduced their rates and one male did not change his rate (Fig. 7). Of the 16 singers who had decreased their song rate during the broadcast, 12 increased their song rates once the stimulus was removed, only three reduced their rates, and again one displayed no change.

After removing one outlier, the distributions of the rate change variables were tested for normality, and the hypothesis of non-normality was rejected (Kolmogorov–Smirnov: difference before and during playback, *p* = 0.146; difference during and after playback *p* = 0.094; difference before and after playback *p* = 0.20). Using two-tailed probabilities for ‘before vs. during’, the change in mode rates was non-significant (*t* = 0.589, *p*. 0.56). The rate change ‘during vs. after’ was highly significant (*t* = − 3.054, *p* = 0.004). The rate change ‘before vs. after’ approached significance (*t* = − 1.788, *p* = 0.082). After correcting probabilities by following a sequential Holm-Bonferroni procedure, these probabilities became for ‘before vs. during’, ‘during vs. after’, and ‘before vs after’, respectively, *p* = 0.56, *p* = 0.012, and *p* = 0.164.

We also performed a one-way ANOVA with repeated measures for the two groups of males that had either increased or decreased their song rates during the broadcast interval. For the 21 males that had increased (or held, *n* = 3) their song rates during the broadcast interval, this dependent variable was normally distributed at each time point—before, during, and after—as assessed using the Shapiro–Wilk’s test (*p* > 0.05). The mean song rates for these males increased from 65.8 ± 13.8 songs/30 s before playback to 70.4 ± 13.3 songs/30 s during playback, which represented a statistically significant increase of 4.7 (95% CI, 2.1 to 7.3) song rate/30 s, *p* < 0.0005. The mean song rate increased again to 72.3 ± 13.4 songs/30 s after playback was discontinued, which represented an increase of 1.9 in song rate/30 s, *p* = 0.39 (95% CI − 1.2 to 4.9) (Fig. 7).

For the 16 males that had decreased their song rates during the broadcast interval, the playback evoked statistically significant changes in the song rate: *F* (2,30) = 18.394. The song rate for these males decreased from 72.9 ± 17.3 songs/30 s before playback to 63.3 ± 20.6 songs/30 s during playback, which represented a statistically significant decrease of 9.6 songs /30 s (95% CI 13.9–5.3) *p* < 0.0005. After the playback stopped, the rate increased to 69.0 ± 17.2, a rebound of 5.7 song rate/30 s (95% CI 0.6–10.8), *p* = 0.026.

## Discussion

### Song rates and rivalry

Within choruses of singing males, many acoustic insects and anurans maintain a minimum distance from their neighbours, resulting in an exclusive space occupied by their song, even while they are not defending some physical resource. Song rate is clearly related to spacing in *S. sphagnorum*. One line of evidence comes from song interactions between neighbour pairs in the field, where we found large differences in song rates for males separated by distances < 4 m, but not for males separated by larger distances. Such observations imply males are listening to each other and are consistent with singer spacing mediated by song. This is supported by results of our speaker broadcast experiments testing how a calling ‘focal’ male would react to a high intensity song indicative of a close-by neighbour.

The results indicate that two sorts of males exist within the population, with some males increasing, others decreasing their song rate. We can only speculate regarding the reason(s) for these differences in singing behaviour of males. The history of individuals involved in the field playback was unknown, including their age, mating status, prior song or condition. Additionally, stored memory about prior inter-neighbour interaction or perceived information about acoustic signals ahead of behavioural tests can have substantial effects (e.g., Bailey and Zuk 2009). Whatever the reasons for the observed differences in acoustic responses, they are consistent with the data of interacting neighbours of Fig. 4b, and showed that acoustic cues alone are sufficient to engender male interactions. What was not seen is also relevant here: during these playbacks there was never any displacement of the focal male in the direction of the speaker, i.e., there was no aggressive escalation, such as occurs in some other katydids (e.g., *Orchelimum* Morris 1971, 1972). This is consistent with a very moderate gain: what they contend for does not merit escalation.

### Spacing with randomness

As a further test of this species’ song functioning in maintaining a distance to a neighbour, we anticipated finding regularity in the spacing of males, as reported in studies conducted with other singing katydids (*Mygalopsis marki*, Thiele and Bailey 1980, Römer and Bailey 1986; *Panacanthus pallicornis* Chamorro-Rengifo et al. 2007; *Conocephalus nigropleurum* and *Orchelimum gladiator* (Table 1), Morris 1967). But our analysis of *S. sphagnorum* spacing in the Niblock population indicated a random distribution, and the spacing within clumps at Trewartha was also random. If males are using song to maintain a minimum distance to a neighbour, why randomness instead of the expected regularity?

Random or regular spacing is also affected by the density of singing males in a chorus. An illustrative empirical example is the genesis of a male chorus of the meadow katydid *Orchelimum gladiator* followed through a season (Morris 1967). The first three maps at low densities indicated no significant departure from randomness. But as the number of males within the population steadily increased the Clarke Evans R ratios indicated significant regularity. The randomness within Niblock or within the clumps at Trewartha is consistent with low densities of singer aggregates.

Spacing within a chorus is also affected by the distribution of preferred singing sites, which could have been the main determinant of the randomness of *S. sphagnorum* spacing. In the katydid studies of *M. marki, P. pallicornis, O. gladiator* and *C. nigropleurum* cited above, males could choose their preferred singing sites in an almost homogeneous vegetation. In the bog substrate used by *S. sphagnorum* the stunted spruce trees vary from bushy to scrawny, short to tall, old to young, isolated to clumped. They stand among an understorey of multiple woody-stemmed shrubs (Ericaceae) and hummocks of sphagnum. All of these plants represent potential singing sites for male *S. sphagnorum*. They sing from spruce trees (trunk, branch, or terminal shoot) and favour heights which reduce the amount of excess attenuation of the high sonic and ultrasonic song frequencies. But they also perch on the low shrubs and may even sing standing on the sphagnum moss surface. When spruce trees are clumped, as in several places at Trewartha, they create, via their intermingled branches a ‘three-dimensional volume’ of rather homogenous vegetation within which multiple perches are available for males. Therefore, tree distribution certainly influences singer distribution, but males could still choose between several available singing options and keep a certain distance to a neighbour based on his perceived song.

### Favoured broadcast positions

In studies in which the position of males within vegetation layers has been studied, males have demonstrated a tendency to call from elevated positions to avoid or reduce the effects of excess attenuation through scattering (Rheinlaender and Römer 1986; Römer and Lewald 1992; cicadas: Doolan and MacNally 1981; moths: Gwynne and Edwards 1986; katydids: Dadour and Bailey 1979; Römer and Bailey 1986; Arak and Eiriksson 1992; crickets: Paul and Walker 1979). The extent to which the broadcasting height influences the active space of a signal has been measured in the katydid *M. marki*, individuals of which call from the top of heath-like scrub plants, allowing them to double this space as compared to singing within the scrub (Römer and Bailey 1986). For *S. sphagnorum*, the uneven, hummocky sphagnum surface represents a transmission environment with strong excess attenuation effects. Using a “biological microphone” approach (Rheinlaender and Römer 1986; Römer and Bailey 1986) to study maximal hearing distances in the bog, we found that males singing from an average perch height of about 60 cm increase their broadcast range threefold compared to the range used when singing from the ground level (Römer and Morris, unpublished results). Within these trees, heights greater than those used by the insects would have been available (Fig. 3), but would have given the insects only a minute, additional advantage in broadcast range.

The observed broadcast height may also represent a trade-off between optimally broadcasting the signal and avoiding predation, because a male calling from the top of the vegetation becomes an easy target for visually hunting birds during the day. However, even though intensive field work was conducted with this insect over many seasons, we have observed no predation events. Therefore, we would favour another interpretation for the reduced singing height in the trees. An essential part of the female’s route to the singing male is on the trunk of the tree; since they must climb up to the singer to mate (all matings observed over the years were up in trees). Male competitors positioned near the base of the tree could intercept females as an alternative strategy. Thus, the resident male must sing high enough to maximize the broadcast range, but not so high that he is unable to defend the base of the trunk effectively. A study of two *Cyphoderris* species (*C. buckelli* and *C. monstrosa*) revealed the difference between the ‘trunk effect’ and its absence (Morris et al. 2002). Male *C. buckelli* interact acoustically in the understorey at high densities and, just like *S. sphagnorum*, respond to the calls of neighbours. However, they do not escalate their acoustic interactions, and it appears that the substrate affords no defensible loci for broadcasting songs. In the same habitat, *C. monstrosa* males defend mature ponderosa pine or lodgepole pine tree trunks in violent fights (Mason 1996).

Females—being flightless—must perform phonotaxis toward the call of a conspecific male, moving over an extremely uneven bog surface covered with strongly sound-attenuating vegetation. Although little is known about the females’ movements, many field matings have been observed, all in trees. Furthermore, lone females without males are frequently seen atop short (< 1 m) trees, giving the impression that they may climb up from the bog to listen for male calls. Such behaviour was predicted by Rheinlaender and Römer (1980) for another katydid (*Tettigonia viridissima*) when they found that directional cues for sound localization can be completely lost in dense vegetation, although female receivers can still hear the stimuli down in the vegetation. Only by climbing up could directional information be regained. Thus, whereas male *S. sphagnorum* use and defend small spruce trees to optimize broadcasting conditions, female receivers may use the same habitat resources to localize males.

### Nearest neighbour distances and acoustic cues for spacing

The mean NNDs differed in the three populations from 5.1 m at Niblock to 6.4 and 8.4 m in two different seasons at Trewartha. Several reasons may account for such differences. Previous studies (Morris 1967; and those reviewed in Gerhardt and Huber 2002) indicate the minimum distance between signalling animals decreases with increasing chorus density. It may be that density was higher at Niblock than at Trewartha (because we did not map the Trewartha populations we cannot give values for density there). However, other factors like different transmission characteristics at the time when the populations were studied cannot be excluded. The reported song transmission experiment was performed during the season when the population revealed a NND of 6.4 m. Furthermore, the literature provides cases where for the same katydid in similar habitat mean NNDs can differ by a factor of two, because of differences in loudness of signalers.

Concerning the maintenance of absolute vs. relative distances to neighbours it is highly unlikely that acoustic insects maintain absolute distances. To judge the absolute distance to a source based on acoustic structure and/or frequency content, a receiver has to determine how the signal has changed during propagation. A prerequisite is knowledge about the signal at the source and about signal degradation/attenuation on the transmission channel (for review see Naguib and Wiley 2001). However, individual signalers may vary in their absolute loudness, but also in the SPL of the signal broadcast into the dorsal or ventral direction of a singing male, which can differ by more than 10 dB (note the relatively high variation of the perceived SPL from a neighbour in Fig. 4a). The receiver has no information about these variables of signalers, nor does he know the height of broadcast position, or the type/density of vegetation and thus excess attenuation properties. In the katydid *M. marki* size and thus broadcast SPL of their calls varies by 7 dB in different populations. Yet, although the mean inter-male distances in the two populations were significantly different (6.2 and 11.5 m), the SPL as perceived from their neighbours did not (Römer and Bailey 1986). This is clear indication for relative, rather than absolute distance estimation.

### Acoustic cues for spacing

What kind of reliable information about the proximity of rival males could be used? In *S. sphagnorum*, the perceived SPL of a neighbour at the mean NND varied between about 55–62 dB, but for shorter distances, it increased considerably to values of up to about 75 dB SPL at 2 m. This increase is associated at the behavioural level with larger differences in the song rate (Fig. 4b). However, in addition to the absolute level of the perceived song of a neighbour, potential information about distance may also be encoded in the relative SPL of the audio compared to ultrasonic modes.

Due to the frequency-dependent, excess attenuation reported for several diverse taxa that communicate with sound, but particularly insects, high-audio and ultrasonic frequencies suffer from stronger attenuation than lower frequencies (Fig. 5; Marten and Marler 1977; Michelsen 1978; Wiley and Richards 1982; Keuper and Kühne 1983; Römer 1998). Compared to almost all other katydids, in which all song elements show the same spectral energy, in *S. sphagnorum* such higher and lower frequencies are distributed in two song modes which are alternated over time in a highly redundant song. The ultrasonic mode is up to 7 dB more intense at shorter distances, but—due to stronger excess attenuation—the audio mode is more intense at distances larger than 6 m. Thus, a receiver that could assess the relative amplitudes of these two song modes separately would gain more detailed information about the proximity of a neighbour compared to a receiver that could measure the overall SPL alone. Degradation of the signal in the time domain can also be used to estimate the distance to a signaler (Morton 1986; Naguib and Wiley 2001). Sources for temporal distortions are reverberations and temperature fluctuations on the transmission channel, but given the structure of the bog, and the elevated positions of most males for singing, relevant reverberations are unlikely. Temperature gradients do exist (see below); but the extremely repetitive song produced over several hours would allow compensating for the occasional loss of the fine temporal song structure. The important information about the song rate, as expressed in the continuous frequency modulation, is maintained over distances much larger than the inter-male distances. Whether and how the nervous system encodes this information is the subject of the second paper in this series (Kostarakos and Römer, this issue).

### The ambiguousness of song rate as sole information during song interactions

Our experimental results support the hypothesis that song rate is an important parameter in agonistic interactions. However, temperature has also been demonstrated to have a profound effect on acoustic signalling in these ectotherm insects, and specifically on the rate with which song elements are produced (Walker 1962; Doherty 1985; Gerhardt and Huber 2002; Symes et al. 2017). As our measurements in the bog showed (Fig. 6), daytime temperatures can fluctuate widely over short periods of time and at different elevations at the same location. The temperatures of the layers of air that are near ground level are largely determined by the intensity of sun radiation, with the highest temperatures occurring where radiation is absorbed by vegetation (i.e., the bog surface). Similarly strong correlations between temperature and height above the ground have been measured in a grassland habitat with vegetation about 30–40 cm high, with the maxima occurring at the top of the vegetation (Römer 2001).

Such variations in temperature may complicate song interactions between male *S. sphagnorum*, due to the strongly linear relationship between song rate changes and temperature (Fig. 4b). The steepness of the linear regression predicts that a male calling from near the ground at an ambient temperature (i.e., 5° higher compared to an elevated position) would call at a song rate about 15–20/30 s higher than his rival, calling from up in a small spruce tree. Because of this song rate ambiguity, varying both due to differences in the temperature and behavioural responses to rivals, males should use additional information about the proximity of rival males. *S. sphagnorum* individuals also sing after sunset and at night when temperatures are generally lower, and presumably temperature gradients at different heights above the ground are either less pronounced or even reversed because of temperature inversions (the latter has potential effects on the active space of the signal, see van Staaden and Römer 1997). Consequently, the information provided by song rate would only be subtly affected by this abiotic parameter and more consistently by the internal state of the sender. Thus, comparable measurements at night are badly needed.

### Potential of masking interference by *C. fasciatus* song

In contrast to species-rich habitats like tropical rainforests, where many species of acoustic insects compete for access to the acoustic communication channel, boreal bogs provide a rare situation in which only two katydid species communicate at the same time. Nevertheless, evidence exists that strong masking interference can occur even in the presence of only two interacting species, if they use spectrally similar and overlapping songs. This can result in the complete suppression of the calling activity of one species by the other or a shift in the diurnal calling activity of one species (Greenfield 1988; Römer et al. 1989; for a review of the masking problem in insects, see Römer 2014).

In the case of song interaction between *S. sphagnorum* and *C. fasciatus*, the potential for masking interference is severe, given the high SPL of a *C. fasciatus* song. It is also unique because the ultrasonic spectrum of *C. fasciatus* overlaps only with the ultrasonic mode of *S. sphagnorum* and not its audio part (Fig. 1). Thus, even when the representation of the ultrasonic mode is completely masked in receivers, the audio mode is not, so that important information about the rate of mode changes will be maintained. Evidence of support for this hypothesis comes from the results of an inspection of the distribution of both species in the Trewartha population (Fig. 2): in the eastern part of the map, a dense accumulation of *C. fasciatus* singers was found (mean inter-male distance as short as 2.5 m) to be interspersed by singers of *S. sphagnorum*. However, in the western part of the mapped population, *C. fasciatus* males were completely absent, but the distribution of *S. sphagnorum* did not differ statistically in both areas. If masking interference were a problem near singing *C. fasciatus* males, we would have expected males of *S. sphagnorum* to have moved towards the western part of the distribution to be free of acoustic competition. Our results lead us to conclude that *S. sphagnorum* escapes the masking interference with the separation of two spectra in two different song modes. However, the masking problem may be different for male and female *S. sphagnorum*. Flightless females have to approach singing conspecific over the bog surface, where *C. fasciatus* males sing at high densities. They experience therefore a higher masking signal, and a reduced signal-to-noise-ratio compared to males singing from more elevated positions. The proximate neuronal mechanism for the escape from masking is described in the second paper of the series (Kostarakos and Römer, this issue).

## Electronic supplementary material

Below is the link to the electronic supplementary material.

S1 Slow-motion video of the movement of the forewings of a male S. sphagnorum while producing the audio and ultrasonic mode of song (Courtesy of Fernando Montealegre-Z). (MP4 1705 KB)

S2 Photograph of a typical section of the Trewartha site. Note the bog substrate used by *S. sphagnorum* consists of stunted spruce trees varying in height, and are isolated to clumped. They stand among an understorey of multiple woody-stemmed shrubs (Ericaceae) and hummocks of sphagnum. All of these plants represent potential singing sites for male *S. sphagnorum*. (JPG 1609 KB)

## Abbreviations

SPL: Sound pressure level
NND: Nearest neighbour distance
