# Supplementary figures and images for: Listening in the bog: I. Acoustic interactions and spacing between males of Sphagniana sphagnorum
Source: J Comp Physiol A Neuroethol Sens Neural Behav Physiol. 2018 Feb 13;204(4):339–51. doi: 10.1007/s00359-018-1250-8 (PMC5849662; doi:10.1007/s00359-018-1250-8)

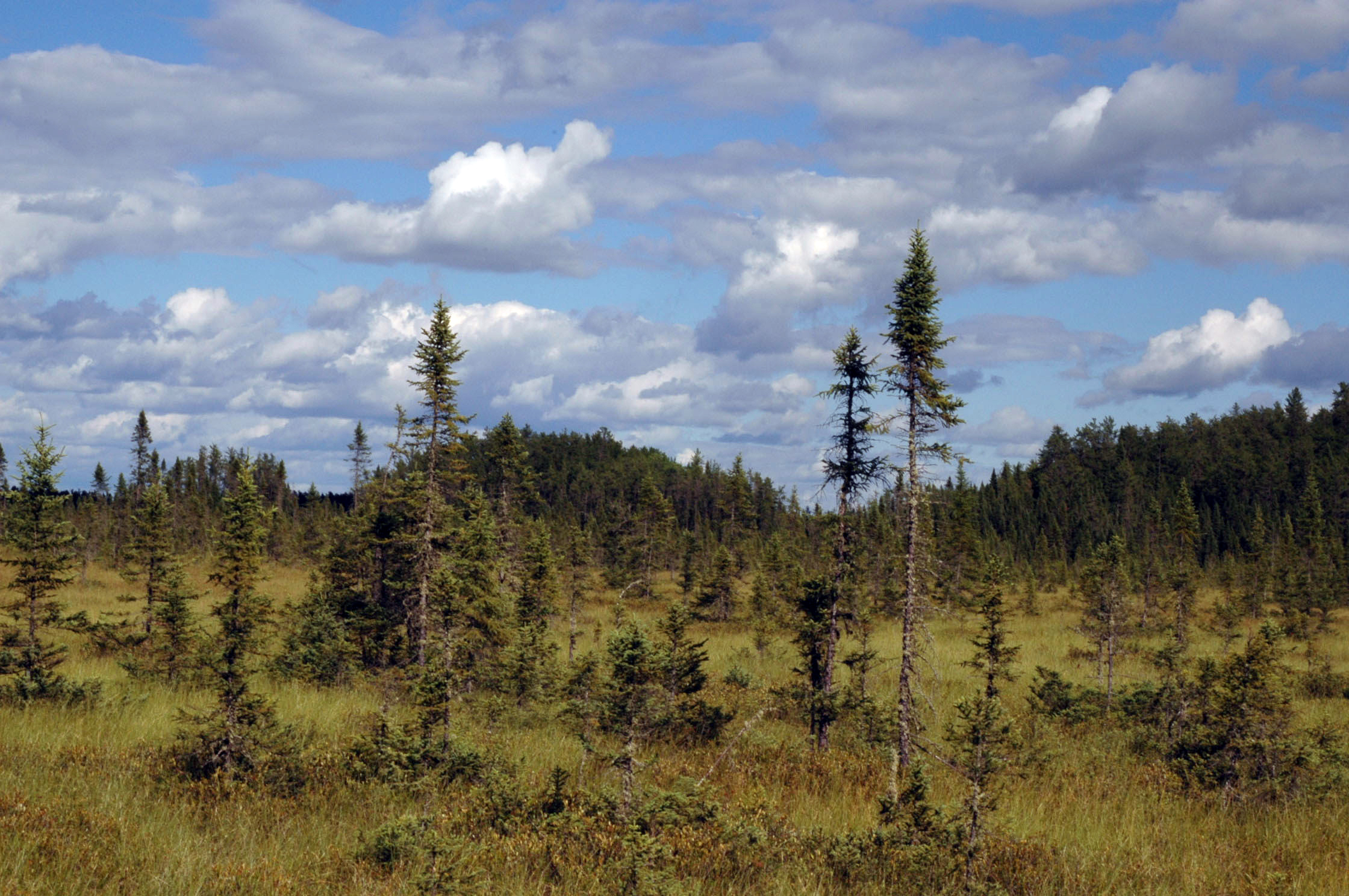

Supplement: Supplementary file 2 — S2 Photograph of a typical section of the Trewartha site. Note the bog substrate used by S. sphagnorum consists of stunted spruce trees varying in height, and are isolated to clumped. They stand among an understorey of multiple woody-stemmed shrubs (Ericaceae) and hummocks of sphagnum. All of these plants represent potential singing sites for male S. sphagnorum. (JPG 1609 KB) [file 359_2018_1250_MOESM2_ESM.jpg]
